# Grain-yield stability among tropical maize hybrids derived from doubled-haploid inbred lines under random drought stress and optimum moisture conditions

**DOI:** 10.1071/CP17348

**Published:** 2018-07-04

**Authors:** Julius Pyton Sserumaga, Yoseph Beyene, Kiru Pillay, Alois Kullaya, Sylvester O. Oikeh, Stephen Mugo, Lewis Machida, Ismail Ngolinda, Godfrey Asea, Justin Ringo, Michael Otim, Grace Abalo, Barnabas Kiula

**Affiliations:** A National Agricultural Research Organisation, National Crops Resources Research Institute, Namulonge, PO Box 7084 Kampala, Uganda; B International Maize and Wheat Improvement Center (CIMMYT), ICRAF House, UN Avenue, Gigiri, Village Market, PO Box 1041-00621, Nairobi, Kenya; C Monsanto, 2 Vermeulen Straat, Petit, 1512, South Africa; D Mikocheni Agricultural Research Institute, PO Box 6226, Dar es Salaam, Tanzania; E African Agricultural Technology Foundation (AATF), PO Box 30709-00100, Nairobi, Kenya; F Ilonga Agricultural Research Institute, PO Box 33, Kilosa, Morogoro, Tanzania

**Keywords:** correlation, East Africa, G-E interaction, heritability, management

## Abstract

Drought is a devastating environmental stress in agriculture and hence a common target of plant breeding. A review of breeding progress on drought tolerance shows that, to a certain extent, selection for high yield in stress-free conditions indirectly improves yield in water-limiting conditions. The objectives of this study were to (*i*) assess the genotype × environment (GE) interaction for grain yield (GY) and other agronomic traits for maize (*Zea mays* L.) across East African agro-ecologies; and (*ii*) evaluate agronomic performance and stability in Uganda and Tanzania under optimum and random drought conditions. Data were recorded for major agronomic traits. Genotype main effect plus GE (GGE) biplot analysis was used to assess the stability of varieties within various environments and across environments. Combined analysis of variance across optimum moisture and random drought environments indicated that locations, mean-squares for genotypes and GE were significant for most measured traits. The best hybrids, CKDHH1097 and CKDHH1090, gave GY advantages of 23% and 43%, respectively, over the commercial hybrid varieties under both optimum-moisture and random-drought conditions. Across environments, genotypic variance was less than the GE variance for GY. The hybrids derived from doubled-haploid inbred lines produced higher GY and possessed acceptable agronomic traits compared with the commercial hybrids. Hybrid CKDHH1098 ranked second-best under optimum-moisture and drought-stress environments and was the most stable with broad adaptation to both environments. Use of the best doubled-haploids lines in testcross hybrids make-up, well targeted to the production environments, could boost maize production among farmers in East Africa.

## Introduction

In East Africa, maize (*Zea mays* L.) is widely used as a major staple. Total maize production for the last 10 years was 236.65 Mt harvested from a total area of 147.17 Mha (FAOSTAT 2014). More than half of the maize produced in East Africa is traded in the commercial maize market, and it makes a major contribution to the economy. Although maize is considered a non-traditional export crop in Africa, its exports from East Africa amounted to 4.71 Mt, valued at US$1.72 million in the last 10 years (FAOSTAT 2014). However, most farmers are unable to attain the potential yield of hybrid maize, owing to biotic and abiotic factors. Among the abiotic factors, drought stress is ranked number one.

In the last decade in Africa, drought stress resulting from climate change has become a major constraint that has gained prominent attention because of its effects on maize productivity (Msowoya *et al*. 2016). In East Africa alone, 34 drought events were reported, affecting >67.8 million people (EM-DAT 2016). Hence, drought remains a major catastrophe causing much stress and suffering to humanity.

Most places in the maize-producing regions of East Africa experience frequent droughts that often coincide with the flowering period of the maize crop, leading to poor grain yields or total crop failure (Omoyo *et al*. 2015). Drought has therefore become a major threat to food security for smallholder farmers who grow maize in drought-prone areas of East Africa. The most economically viable and sustainable option for alleviating the situation is breeding and deploying improved drought-tolerant, high-yielding maize cultivars for the farmer to assure profitable yields even in drought years (Cairns *et al*. 2013). This has called for the identification of different sources of drought-tolerance traits and an efficient means of generating new products to cope with the situation. One of the ways is the use of doubled-haploid technology in rapid-development, drought-tolerant lines. Application of doubled-haploid technology has made possible the development of inbred lines in one or two generations (Prigge *et al*. 2011) compared with classical pedigree methods that produce 96.9% homozygous lines after six to 10 generations of selfing heterozygous material (Hallauer *et al*. 2010). This, in turn, has enabled rapid development of hybrids from doubled-haploid lines that are drought tolerant. These hybrids will improve food security for smallholder farmers in many areas of Africa.

The identification of genotypes with a high yield potential, coupled with wide adaptability and stability, is a key target of any maize-breeding program (Mendes *et al*. 2012). The major drawback, however, in the selection of genotypes with high yield potential in different environments is the genotype × environment (GE) interaction, because strong interactions can hamper the selection process. Thus, genotypes that perform well in one environment may not do as well in another (Mendes *et al*. 2012). GE interactions have been investigated through the use of statistical tools such as additive main effects and multiplicative interaction (AMMI) analysis for grain yield and grain micronutrient concentrations and stability (Oikeh *et al*. 2004; Gauch 2006); and genotype main effect plus GE interactions (GGE) (Yan 2001) for the analyses of grain yield and stability in tropical maize. Several studies have been undertaken to assess the performance of genotypes under different environmental conditions (Beyene *et al*. 2013). However, most of these studies were carried out in the same country. Collaborative efforts among research organisations in five countries, Kenya, Mozambique, South Africa, Tanzania and Uganda, have developed quite a diverse germplasm base through the Water Efficient Maize for Africa (WEMA) project. The WEMA project is a public–private partnership established to develop drought-tolerant and insect-protected maize by using conventional breeding, marker-assisted breeding and biotechnology, with a goal to make these varieties available royalty-free to smallholder farmers in Sub-Saharan Africa through African seed companies (Oikeh *et al*. 2014; Edge *et al*. 2018). Materials developed are tested in multiple environments that represent drought-stress and non-stress (optimum-moisture) locations to identify high-yielding and adapted varieties for release and cultivation in the respective countries. With the formation of the East African Community, seed companies prefer licensing and marketing maize seed for varieties that have been commercialised in more than one country. The tremendous progress made in WEMA product developments has led to gains in grain yield performance as reported in Maize Regional Trials (Sserumaga *et al*. 2016; Beyene *et al*. 2017). With the changing climatic conditions, information and utilisation of knowledge on the genetic performance of different germplasm are paramount in a breeding program. The WEMA project extensively used doubled-haploid technology in inbred line development, which constituted most of the maize hybrids that were to be tested in multiple environments among WEMA countries before commercialisation.

The objectives of this study were to (*i*) assess GE interaction for grain yield and other agronomic traits in maize across East African agro-ecologies; and (*ii*) evaluate agronomic performance and stability in Uganda and Tanzania under optimum and random drought conditions.

## Materials and methods

### Genetic materials and performance evaluation

In total, 43 hybrids derived from doubled-haploid inbred lines were selected for this study (see Supplementary materials table 1, available at the journal’s website). The 43 hybrids were selected based on grain yield, disease resistance and other agronomic traits from previous preliminary yield testing. Sufficient seed of the 43 hybrids was produced in 2013 at the Kenya Agricultural and Livestock Research Organisation (KARLO) Maize Research Station at Kiboko, Kenya. Each female parent was planted in five rows of 5 m length, and the male donor plants were planted at two different times (–5 and 0 days) to effect nicking at pollination and synchronise flowering. All recommended agronomic practices for maize production for the agro-ecologies were applied (Sserumaga *et al*. 2016). Immediately before flowering, all of the ears of the female plants were covered with shoot bags. During hand pollination, pollen was collected and bulked from the male plants when 20% of the males had started to shed pollen. The 43 hybrids along with seven checks were evaluated across eight sites in Uganda and five sites in Tanzania (Supplementary materials table 2). Optimum-moisture sites were selected based on the total amount of precipitation and the trials were established during the main season, whereas at the drought-stress sites, trials were established so that the stress coincided with 2–3 weeks before 50% flowering in order to have sufficient drought stress for evaluation of the materials. The experimental design was a 5 × 10 α-lattice with two replications at each location. An experimental unit was a two-row plot, 5 m long, spaced 0.75 m between rows and 0.25 m between plants. Two seeds were planted per hill and subsequently thinned to one plant per hill at 4 weeks after emergence, to give a final plant population density of 53 333 plants ha^−1^. In all the experiments, standard agronomic and cultural practices including weeding and appropriate fertiliser applications were followed.

**Table 1:** Mean-squares and degrees of freedom fromANOVAfor grain yield and agronomic traits of 43 testcross hybrids, two internalWEMAhybrid checks, three commercial hybrids and two local hybrid checks across drought, optimum conditions, and environments in Uganda and Tanzania

Source	d.f.					Mean-square						
		Grain yield(t ha^–1^)	Days to anthesis	Anthesis–silking (days)	Plant height (cm)	Ear height (cm)	Ear position (cm)	No. of ears per plant	Grain moisture (%)	Husk cover (%)	Ear aspect (1–5)	Plant aspect (1–5)
*Random drought*												
Environment	2	1181.17***	17006.71***	4.71*	2080.12*	5628.61***	0.10***	1.28***	24.57***	25.97**	49.97***	46.74***
REP (ENV)	3	21.76***	22.69n.s.	17.05***	271.76n.s.	363.91n.s.	0.00n.s.	0.01n.s.	21.69***	125.34***	0.91**	1.27***
Genotype	49	2.67*	15.10n.s.	2.51n.s.	456.13n.s.	148.51n.s.	0.01n.s.	0.03n.s.	3.40n.s.	7.77**	0.49***	0.18n.s.
GEI	98	1.84n.s.	13.26n.s.	1.87n.s.	445.37n.s.	121.63n.s.	0.01n.s.	0.02n.s.	2.48n.s.	6.81*	0.33**	0.17n.s.
Error	147	1.84	12.68	1.88	545.49	201.87	0.01	0.03	3.34	4.46357	0.21	0.15
*Optimum moisture*												
Environment	9	105.06***	1953.03***	307.92***	46815.35***	7337.39***	0.08***	1.07***	133.07***	72.74***	16.73***	11.80***
REP (ENV)	10	7.95***	21.38***	1.26n.s.	1267.09**	1138.12**	0.01n.s.	0.14n.s.	4.93***	3.42n.s.	0.57***	0.60***
Genotype	49	3.73***	8.79***	0.87n.s.	873.03**	530.05*	0.01n.s.	0.11n.s.	4.13***	7.01**	0.65***	0.25*
GEI	441	1.67***	4.99n.s.	0.96n.s.	567.60*	396.09n.s.	0.01n.s.	0.13n.s.	1.74*	4.61n.s.	0.23***	0.23**
Error	488	1.19	4.84	0.99	487.44	372.73	0.01	0.12	1.49	4.21	0.16	0.18
*Across environments*												
Environment	12	314.84***	4612.58***	278.26***	49491.46***	7359.54***	0.09***	1.28***	105.90***	76.22***	21.09***	1356.16***
REP (ENV)	13	11.14***	21.71***	5.21***	1086.12*	997.35***	0.01n.s.	0.11n.s.	8.80***	23.74***	0.65***	105.38***
Genotype	49	4.47***	11.12**	1.17n.s.	832.07**	481.4339*	0.01n.s.	0.09n.s.	5.19***	9.17***	0.91***	90.06***
GEI	588	1.72**	7.16n.s.	1.24n.s.	548.2n.s.	349.11n.s.	0.01n.s.	0.10n.s.	1.91n.s.	4.90*	0.25***	23.70n.s.
Error	635	1.34	6.75	1.19	498.01	341.61	0.01	0.1	1.92	4.26	0.17	21.38

GEI, Genotype × environment interaction. *P < 0.05; **P < 0.01; ***P < 0.001; n.s., not significant

**Table 2:** Mean performance of the top 15 hybrids and bottom five hybrids across different drought and optimum environments in Uganda and Tanzania

Rank	No.	Genotype	GY (t ha^−1^)	AD	ASI	HC (%)	EA (1–5)	EH (cm)	EPP	EP (cm)	MOI (%)	PA (1–5)	PH (cm)
				(days)									
*Across three drought environments*													
**Top**													
1	G14	CKDHH1090	7.01	63.39	1.83	4.38	2.43	95.43	0.47	0.97	18.24	2.74	203.09
2	G16	CKDHH1098	6.82	64.60	2.56	3.88	2.78	112.21	0.54	0.91	18.87	2.78	200.81
3	G6	CKDHH1075	6.47	64.66	2.68	4.29	2.89	103.19	0.50	0.97	17.24	2.82	204.67
4	G43	CKDHH1044	6.47	64.09	2.43	5.22	2.89	96.46	0.48	0.91	18.37	2.65	198.60
5	G32	CKDHH0959	6.44	63.71	2.41	5.25	2.91	103.56	0.51	1.00	18.47	2.71	198.12
6	G30	CKDHH0954	6.30	65.62	2.63	4.97	2.79	94.89	0.47	0.89	18.20	2.72	195.09
7	G44	CZH0616	6.29	60.99	3.37	4.81	2.39	101.61	0.50	0.91	16.63	2.72	200.03
8	G22	CKDHH1148	6.26	64.80	2.59	5.47	2.69	107.54	0.50	0.92	17.92	2.76	210.22
9	G23	CKDHH1132	6.12	67.22	2.24	6.82	3.12	102.04	0.65	0.90	17.81	2.84	173.03
10	G33	CKDHH0960	6.10	67.63	3.40	6.49	3.22	104.77	0.51	0.86	18.81	3.23	206.67
11	G9	CKDHH1081	6.04	62.73	2.29	6.73	2.35	107.11	0.49	0.96	16.37	2.59	214.57
12	G3	CKDHH1068	5.99	64.92	2.09	9.00	2.86	97.69	0.48	0.97	17.41	2.97	201.71
13	G4	CKDHH1070	5.91	63.75	3.33	6.77	2.90	103.80	0.49	0.91	18.04	2.79	209.29
14	G19	CKDHH1106	5.87	63.23	2.96	5.36	2.45	96.18	0.45	0.91	18.28	2.58	208.47
15	G39	CKDHH1007	5.86	64.45	2.21	7.81	2.69	106.96	0.55	0.95	18.18	2.57	194.67
**Bottom**													
45	G42	CKDHH1143	5.03	65.74	2.54	6.14	2.92	98.73	0.50	0.79	17.71	2.84	201.52
46	G12	CKDHH1088	4.90	65.45	2.77	5.13	2.52	111.67	0.54	0.84	18.53	3.05	210.46
47	G27	CKDHH1145	4.89	65.34	3.40	10.96	2.58	102.29	0.55	0.99	18.54	2.47	199.62
48	G48	Com Check 3	4.86	64.58	3.02	8.82	2.65	103.37	0.56	0.79	16.83	2.78	187.18
49	G10	CKDHH1078	4.85	64.52	2.93	3.64	2.44	102.97	0.47	0.93	17.52	2.95	218.48
50	G24	CKDHH1134	4.69	68.62	2.04	8.06	2.58	97.04	0.51	0.87	18.10	2.84	190.67
	Mean		5.64	65.18	2.71	6.05	2.68	103.71	0.51	0.90	17.98	2.81	203.35
	Minimum		4.69	60.99	1.83	3.64	1.91	94.48	0.45	0.78	16.19	2.47	173.03
	Maximum		7.01	68.62	4.14	10.96	3.22	114.88	0.65	1.00	19.31	3.23	234.13
	l.s.d. (*P* = 0.05)		1.14	3.65	1.01	3.18	0.58	15.90	0.12	0.12	1.51	0.39	28.19
*Across 10 optimum environments*													
**Top**													
1	G15	CKDHH1097	7.57	63.70	1.65	5.11	2.81	106.97	0.49	0.95	18.20	2.73	224.89
2	G16	CKDHH1098	7.43	62.76	1.55	4.64	2.88	109.59	0.47	0.94	18.34	2.67	232.20
3	G6	CKDHH1075	7.4	62.30	1.51	4.97	2.61	113.84	0.50	0.96	17.23	2.73	230.24
4	G14	CKDHH1090	7.38	62.72	1.00	4.80	2.40	107.68	0.47	0.96	17.44	2.73	228.35
5	G25	CKDHH1141	7.33	63.56	1.21	4.42	2.76	110.16	0.49	0.95	17.49	2.77	224.18
6	G44	CZH0616	7.31	61.22	1.49	5.29	2.48	106.66	0.49	0.97	17.28	2.60	217.48
7	G32	CKDHH0959	7.29	62.42	1.65	4.93	2.87	109.68	0.49	1.01	18.28	2.73	224.55
8	G22	CKDHH1148	7.28	64.19	1.02	5.15	2.69	108.28	0.48	0.99	17.94	2.69	224.93
9	G43	CKDHH1044	7.15	63.04	1.37	4.35	2.85	109.53	0.50	0.99	17.86	2.63	221.01
10	G28	CKDHH1146	7.09	63.47	1.61	4.36	2.56	116.72	0.51	0.95	17.49	2.62	232.04
11	G34	CKDHH0969	7.09	63.77	1.31	5.05	2.49	110.72	0.48	0.95	18.29	2.76	231.98
12	G9	CKDHH1081	7.07	62.89	1.10	4.94	2.28	111.98	0.50	1.08	16.80	2.60	226.88
13	G38	CKDHH1005	7.05	62.78	1.32	6.26	2.51	113.40	0.48	0.99	18.10	2.73	239.04
14	G39	CKDHH1007	7.00	63.19	1.34	6.11	2.69	106.26	0.49	0.99	17.82	2.71	217.52
15	G45	WE1101	6.98	62.00	1.52	5.74	2.24	106.45	0.51	1.00	18.01	2.62	216.47
**Bottom**													
45	G46	Com Check 1	6.21	61.63	1.41	6.59	2.34	124.40	0.52	1.32	17.65	2.84	236.46
46	G10	CKDHH1078	6.13	62.26	1.62	4.52	2.30	109.46	0.48	0.99	17.50	2.65	230.10
47	G50	Local Check	6.08	63.33	1.55	4.47	2.48	110.54	0.51	0.96	17.64	2.73	223.98
48	G49	Local Check	5.93	62.99	1.64	5.02	2.56	121.51	0.49	1.02	17.37	3.00	243.68
49	G24	CKDHH1134	5.88	64.10	1.20	5.57	2.43	115.12	0.51	0.98	16.99	2.71	221.91
50	G7	CKDHH1076	5.83	62.77	1.33	5.52	2.63	116.68	0.52	1.03	17.12	2.79	222.43
	Mean		6.74	63.05	1.42	5.24	2.60	109.89	0.49	0.99	17.74	2.74	225.34
	Minimum		5.83	61.22	1.00	4.11	2.24	102.9	0.47	0.9	16.8	2.55	214.79
	Maximum		7.57	64.26	1.98	6.71	2.88	124.4	0.52	1.32	18.66	3.00	243.68
	l.s.d. (*P* = 0.05)		0.788	1.41	0.64	1.30	0.28	11.43	0.05	0.23	0.79	0.26	15.21

GY, Grain yield; AD, days to anthesis; ASI, anthesis–silking interval; HC, husk cover; EA, ear aspect (scale of 1–5, where 1 is uniform cobs with the preferred texture and 5 is cobs with undesirable texture); EH, ear height; EPP, no. of ears per plant; EP, ear position; MOI, grain moisture; PA, plant aspect; PH, plant height. Ranking based on yield

### Data collection

Under both drought-stress and optimum-moisture conditions, data collected included: days to anthesis (days from planting to when 50% of plants had shed pollen) and days to silking (days from planting to when 50% of plants had extruded silks); anthesis–silking interval (determined as the difference between days to silking and days to anthesis); plant height (measured in cm as the distance from the base of the plant to the height of the first tassel branch); number of ears per plant (determined by dividing the total number of ears produced per plot by the number of plants harvested per plot); husk cover (obtained by dividing the number of ears with poor husk cover by the number of plants harvested per plot); ear aspect (rated on a scale of 1–5 where 1 is uniform ears with the preferred texture and 5 is ears with the undesirable texture); plant aspect (PA) (rated on a scale of 1–5 where 1 is short plant with uniform and short ear placement and 5 is tall plants with high ear placement ear position); and grain moisture. All of the ears harvested from each plot were weighed and representative samples of ears were shelled to determine the percentage moisture of the grain, using a Dickey–John moisture meter at all locations. Grain yield (t ha^−1^) was calculated from ear weight based on a shelling percentage of 80% and grain moisture content of 12.5%.

### Statistical analyses

#### Analysis of variance and GE interaction under drought and optimum conditions

Individual analyses of variance (ANOVAs) were carried out on the data for each genotype. For the joint analysis of all datasets, we used a mathematical model that considered sites and progenies as random effects and was equivalent to the following equation described by Cruz and Regazzi (1994):

$$Y_{ijk}=\mu +G_{i}+A_{i}+GA_{ij}+\left(B/A_{jk}\right)+\varepsilon_{ijk}$$

where Y_*ijk*_ is observed value of the ith progeny of the *j*th environment in the *k*th replications; μ is general mean; G is effect of the ith genotype (*i* = 1, 2,...i); A is effect of the jth environment (*j* =1, 2,… j); GA is effects of the interaction of the ith progeny with the jth environment; B/A_jk_ is effect of the kth block within the jth environment; and ε_*ijk*_ is random error. ANOVA for all traits was done separately for each environment and combined across locations by using the PROC MIXED procedure from SAS 9.3 (SAS Institute 2011). For the combined analysis, variances were partitioned into relevant sources of variation to test for differences among genotypes and the presence of GE interactions. In the across-environment ANOVA, genotype effects were tested for significance by using the corresponding interactions with the environment as the error term; the GE interaction was tested by using the pooled error.

#### Genotype performance under different conditions

Individual locations or across analysis were computed for grain yield and other traits by using the mixed-model analysis in META-R to generate a best linear unbiased estimate (BLUE) for all genotypes (Alvarado *et al*. 2015). For comparing entries evaluated in different locations, the entry means were expressed as a percentage of the average performance of the best check hybrid at the respective locations.

#### Genetic variances and heritability under different conditions

Estimates of genotypic ($\sigma_{G}^{2}$), location ($\sigma_{L}^{2}$), genotype × location ($\sigma_{G\times L}^{2}$) and error (σ^2^E) variance were calculated using PROC MIXED (option = REML) of SAS. Broad-sense heritability (H^2^) was calculated as the proportion of genetic variance over the total phenotypic variance, and for individual trials was estimated according to Hallauer *et al*. (2010):

$$r_{g}=\frac{r_{p^{\left(12\right)}}}{{\left(H_{1}\times H_{2}\right)}^{1/2}}$$

where $\sigma_{G}^{2}$ is the genotypic variance, $\sigma_{E}^{2}$ is the error variance, and r the number of replications. Broad-sense heritability for traits across environments was estimated by using the variance components according to Hallauer *et al*. (2010) as:

$$Y_{ij}-Y_{J}=\lambda_{1}\xi_{i}1\eta_{j1}+\lambda_{2}\xi_{i2}\eta_{j2}+\varepsilon_{ij}$$

where $\sigma_{G}^{2}$, $\sigma_{G\times L}^{2}$ and $\sigma_{G\times L}^{2}$ are genotypic, genotype × location and residual variance components, respectively; E is the number of environments; and R is the number of replications. Genotypic correlations (*r*_g_) among locations were estimated according to Cooper *et al*. (1996) as:

$$r_{g}=\sigma_{G}^{2}/\left(\sigma_{G}^{2}+\sigma_{ge}^{2}-0.5{\left(\sigma_{g1}-\sigma_{g2}\right)}^{2}\right)$$

where *r*_p(1,2)_ is the phenotypic correlation between the traits measured in locations 1 and 2; and H_1_ and H_2_ are the broad-sense heritabilities for the traits measured in locations 1 and 2, respectively.

#### Genetic correlations among test locations

The genetic correlation among the pairs of environments for each trait under study was obtained as suggested by Yamada (1962), using the following expression:

$$H^{2}=\frac{\sigma_{G}^{2}}{\left[\sigma_{G}^{2}\left\{\frac{\sigma_{E}^{2}}{r}\right\}\right]}$$

where *r*_g_ is coefficient of correlation between the two locations for a certain trait, σ_g1_ is genetic variance at location 1, σ_g2_ is genetic variance at location 2, $\sigma_{g}^{2}$ is joint genetic variance of the joint analysis, and $\sigma_{ge}^{2}$ is variation of the GE interaction.

Cluster analysis using Ward’s minimum variance method (Ward 1963) was performed on group environments based on genetic correlations among the environments, using META-R (Alvarado *et al*. 2015).

#### GGE biplot analysis of grain yield response and stability

Adjusted data on the grain yield from ANOVA were subjected to GGE biplot analysis (Yan 2001) to determine grain yield stability and the pattern of response of genotypes evaluated across the environments. The analyses were done and biplots generated using the GGEbiplot software version 7 (www.ggebiplot.com/biplot). The GGE biplot Model 3 equation used was:

$$H^{2}=\frac{\sigma_{G}^{2}}{\left[\sigma_{G}^{2}+\frac{\sigma_{G\times L}^{2}}{E}+\frac{\sigma_{E}^{2}}{ER}\right]}$$

where Y_*ij*_ is the average yield of genotype *i* in environment *j*; Y_*J*_ is the average yield across all genotypes in environment *j*; λ_1_ and λ_2_ are the singular values for PC1 and PC2, respectively; ξ_*i*1_ and ξ_*i*2_ are the principal component scores PC1 and PC2, respectively, for genotypei; η_*j*1_ and η_*j*2_ are the PC1 and PC2 scores, respectively for environment *j*; and ε_*ij*_ is the residual of the model associated with genotype *i* in environment *j*.

## Results

### ANOVA and GE under drought and optimum moisture conditions

Analysis of variance across three locations revealed that environment was highly significant for all traits, whereas genotype was significant for grain yield, husk cover and ear aspect (Table 1). The GE interaction was significant for ear aspect and husk cover. This implied differences in yield performance among the test materials, although there was no differential response at different locations.

Under conditions of optimum moisture, combined ANOVA across 10 locations revealed that genotype, environment, and GE interaction were highly significant (P < 0.0001) for grain yield (Table 1), suggesting various responses in yield performance among the test materials at different locations. Environment was significant for all the other traits, and genotype was significant for most traits except anthesis–silking interval, ear position and ears per plant (Table 1). The GE interaction was significant only for plant height, grain moisture, ear aspect and plant aspect (Table 1).

Combined ANOVA across all the 13 locations showed that genotype, environment, and GE interaction were all significant for grain yield (Table 1). In addition, environment was highly significant (P < 0.001) for all the traits and genotype was significant for all traits except anthesis–silking interval, ear position and ears per plant (Table 1). The GE interaction was significant for grain yield (P < 0.01), husk cover (P < 0.05), and ear aspect (P < 0.001) (Table 1). This meant that there were significant differences in yield performance among the test genotypes at different locations.

### Hybrid performance under drought-stress and optimum-moisture conditions

Genotype performance across drought-stress environments varied with a grand mean of 5.6 t ha^−1^. The grain yield of the test hybrids varied from 4.7 t ha^−1^ for CKDHH1134 (G24) to 7.0 t ha^−1^ for CKDHH1090 (G14) (Table 2). The highest yielding testcross hybrid (CKDHH1090) had a 44.2% yield advantage over the best commercial hybrid, Com Check 3 (G48). There were variable responses to phenology, with days to anthesis ranging from 61 to 68.6 days, although the anthesis–silking interval values were comparable (1.8–3.4 days) among all testcrosses and the commercial checks (Table 2). Heritability estimates for the different traits were generally very low (0–0.29), except for PA, which had the highest heritability (Table 3).

**Table 3:** Variance decomposition and heritability for grain yield and agronomic traits of 43 testcross hybrids, two internal WEMA hybrid checks, three commercial hybrids and two local hybrid checks across drought, optimum conditions, and environments in Uganda and Tanzania

Random drought								Optimum-moisture							Across environments						
	$\sigma_{G}^{2}$	$\sigma_{L}^{2}$	$\sigma_{G\times L}^{2}$	$\sigma_{E}^{2}$	Mean	l.s.d.	H^2^	$\sigma_{G}^{2}$	$\sigma_{L}^{2}$	$\sigma_{G\times L}^{2}$	$\sigma_{E}^{2}$	Mean	l.s.d.	H^2^	$\sigma_{G}^{2}$	$\sigma_{L}^{2}$	$\sigma_{G\times L}^{2}$	$\sigma_{E}^{2}$	Mean	l.s.d.	H^2^
GY	0.07	11.74	0.09	0.83	5.64	1.14	0.29	0.11	1.03	0.22	1.15	7.07	0.78	0.57	0.11	3.11	0.21	1.06	6.74	0.65	0.66
AD	0.46	185.35	3.08	4.22	65.18	3.65	0.21	0.17	19.91	0.00	4.70	62.37	1.41	0.40	0.15	49.04	0.44	5.01	63.05	1.36	0.38
ASI	0.03	0.00	0.02	0.75	2.71	1.01	0.18	0.00	3.15	0.00	0.97	1.00	0.64	0.00	0.00	2.87	0.00	0.96	1.42	0.55	0.00
PH	0.00	12.10	0.00	406.97	203.35	28.19	0.00	15.88	460.82	32.48	483.49	230.23	15.21	0.34	10.86	487.44	18.20	481.11	225.34	13.35	0.32
EH	0.00	52.86	0.00	129.44	103.71	15.90	0.00	5.19	64.72	17.42	275.10	111.26	11.43	0.23	3.92	66.00	8.56	254.73	109.89	9.68	0.24
EP	0.00	0.00	0.00	0.01	0.51	0.12	0.00	0.00	0.00	0.00	0.01	0.49	0.05	0.00	0.00	0.00	0.00	0.01	0.49	0.04	0.00
EPP	0.00	0.01	0.00	0.01	0.90	0.12	0.09	0.00	0.01	0.00	0.12	1.02	0.23	0.00	0.00	0.01	0.00	0.09	0.99	0.17	0.00
MOI	0.10	0.16	0.00	1.78	17.98	1.51	0.25	0.12	1.31	0.12	1.42	17.66	0.79	0.60	0.14	1.02	0.03	1.54	17.74	0.68	0.70
HC	0.44	0.07	1.04	3.10	6.05	3.18	0.25	0.12	0.67	0.22	3.99	5.08	1.30	0.35	0.19	0.69	0.28	3.88	5.24	1.18	0.51
EA	0.02	0.49	0.06	0.15	2.68	0.58	0.35	0.02	0.16	0.04	0.13	2.57	0.28	0.68	0.03	0.21	0.04	0.14	2.60	0.25	0.76
PA	0.01	0.46	0.01	0.09	2.81	0.39	0.21	0.00	0.11	0.03	0.13	2.71	0.26	0.13	0.00	0.16	0.02	0.12	2.74	0.22	0.35

GY, Grain yield; AD, days to anthesis; ASI, anthesis–silking interval; PH, plant height; EH, ear height; EP, ear position; EPP, no. of ears per plant; MOI, grain moisture; HC, husk cover; EA, ear aspect (scale of 1–5, where 1 is uniform cobs with the preferred texture and 5 is cobs with undesirable texture); PA, plant aspect. σ^2^_G_, σ^2^_G×L_,σ^2^_E_: Genotypic, genotype × location, and residual variance components, respectively: H^2^, heritabilit

Genotype performance varied significantly across optimum moisture environments with a grand mean of 6.71 ha^−1^ (Table 2). The grain yield of the test hybrids varied from 5.8 t ha^−1^ for CKDHH1076 (G7) to 7.6 t ha^−1^ for CKDHH1097 (G15). The highest yielding testcross (CKDHH1097) gave 23% yield advantage over the best popular commercial hybrid, Com Check 1 (G46). All genotypes had comparable maturity, with days to anthesis ranging from 61.2 to 64.2 days. Therefore, all testcross hybrids could be categorised as early-maturing genotypes. Medium to high heritability estimates were found for most traits. The highest heritability of 0.68 was recorded for ear aspect, followed by moisture content (0.60), and the least was for plant aspect (H^2^ = 0.13).

Average performance across all test environments showed a grand mean yield of 5.6 t ha^−1^, but grain yield of the best performing, top 10 test hybrids varied from 4.7 t ha^−1^ for CKDHH10960 (G33) to 7.0 t ha^−1^ for CKDHH1090 (G14) (Supplementary materials table 3). The highest yielding testcross (CKDHH1090) had a 42.9% grain yield advantage over the best popular commercial hybrid, Com Check 3 (G48). The genotypes varied in days to phenological development with days to anthesis ranging from 61.0 to 68.6 days, and entry CZH0616 (G44) with 61 days was considered the earliest maturing genotype. Among the test materials, genotype CKDHH1098 (G16) had the best husk cover of 3.9. Also, heritability estimates were highest for ear aspect (H^2^ = 0.76) (Table 3).

### Genetic variances and heritability under different environmental conditions

Estimates of genotypic, location and genotype × location variances under random drought, optimum moisture conditions and across all test locations are presented in Table 3. Under drought, husk cover was the only factor where the genotypic variance was larger than the location variance. The results showed that environment accounted for 92.2% of the total variation in grain yield. The H2 for grain yield was only 0.29 under drought conditions.

Similarly, under optimum moisture conditions, genotypic and genotype × location variances were smaller than location for all traits. The genotypic variance was larger than the genotype × location variance for days to anthesis alone. The results showed that location accounted for 41% of the total variation for grain yield. The H^2^ for grain yield was moderate, 0.57 under optimum moisture condition.

Across locations, genotypic and genotype × location variances were smaller than the location variance for all traits. The results indicated that genotypic and genotype × location variances accounted for <5%, whereas location accounted for 69.2% of the total variation in grain yield. The H2 for grain yield was 0.66.

### Genetic correlations among test locations

**Fig. 1:**
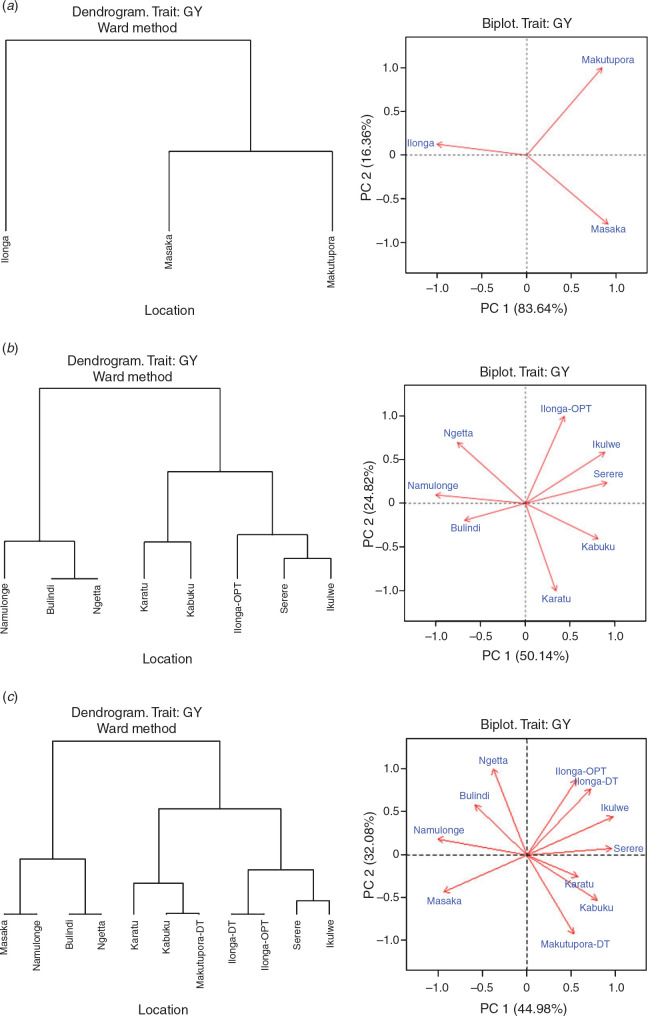
Clustering of (*a*) three locations with significant grain yield under drought conditions, (*b*) eight locations with significant grain yield under optimum conditions, and (*c*) 11 locations based on grain yield, in Uganda and Tanzania.

The genetic correlations among locations were based on grain yield and were used for cluster analysis to classify the environments for their yield potential and stability. Under random drought conditions, genetic correlations among locations were lowest for Ilonga_DT vs Masaka (0.13) and highest for Masaka vs Makutupora_DT (0.6) (Supplementary materials table 4). Clustering based on genetic correlation for grain yield revealed two clusters at 83.64% (Fig. 1a). Cluster I consisted of Ilonga and Cluster II comprised Masaka and Makutupora (Fig. 1a).

Under optimum moisture conditions, genetic correlations among locations were lowest for Abii vs Ikulwe (–0.54) and the highest for Bulindi vs Ngetta (0.39) (Supplementary materials table 4). Clustering based on genetic correlation for grain yield revealed two main clusters at 50.1% (Fig. 1b). Cluster I consisted of three locations that were separated into Namulonge and a subcluster into Bulindi and Ngetta in Uganda. Cluster II consisted of two major subclusters that were divided into subsubclusters.

Across all environments, genetic correlations among locations ranged from –0.54 to +0.58 (Supplementary materials table 4). Clustering based on genetic correlation for grain yield revealed two main clusters at 45.0% (Fig. 1c). Cluster I consisted of two subclusters, the first clustering Masaka and Namulonge and the other Bulindi and Ngetta. Cluster II also had two subclusters, one with Karatu clustered with a sub-subcluster of Kabuku and Makutupora DT, and the other consisting of two sub-subclusters that included Ilonga-DT and Ilonga-OPT and then Serere and Ikulwe, respectively.

### GGE biplot analysis for grain yield response and stability of maize genotypes evaluated across different environmental conditions

**Fig. 2:**
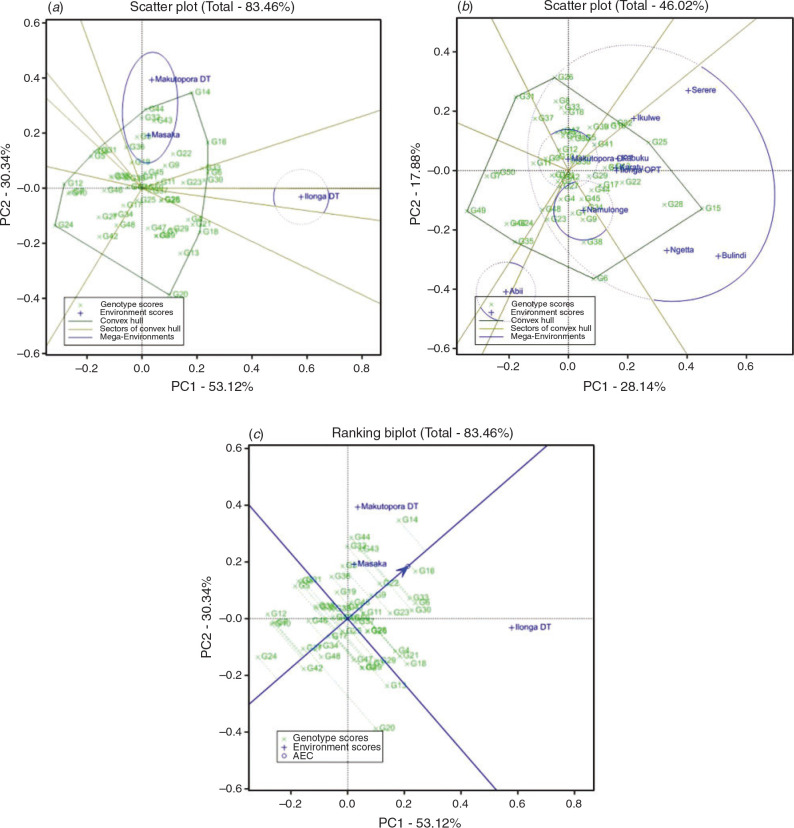
Which-won-where biplot of grain yield of 50 maize varieties evaluated across (*a*) three locations under drought conditions, and (*b*) 10 locations under optimum conditions in Uganda and Tanzania. (*c*) Mean vs stability view of the genotype main effect plus genotype × environment interaction biplot based on yield data of 50 maize varieties evaluated across three locations under drought conditions in Uganda and Tanzania.

The GGE biplot analysis was used to examine the performance of genotypes in the different environments under different conditions. The view of the polygon of the GGE biplot under drought indicated the best genotypes in each environment (Fig. 2a). The presence of two or more environments within a sector shows that a single genotype has the highest yield in those environments. If the environments fall into different sectors, different genotypes performed well in different environments. PC1 explained 53.1% of total variation, and PC2 explained 30.3%. Thus, these two axes accounted for 83.5% of the GGE variation for grain yield under drought (Fig. 2a). For optimum moisture conditions, PC1 explained 28.14% of total variation, whereas PC2 explained 17.88%. Thus, these two axes accounted for 46.02% of the GGE variation for grain yield (Fig. 2b).

**Fig. 3:**
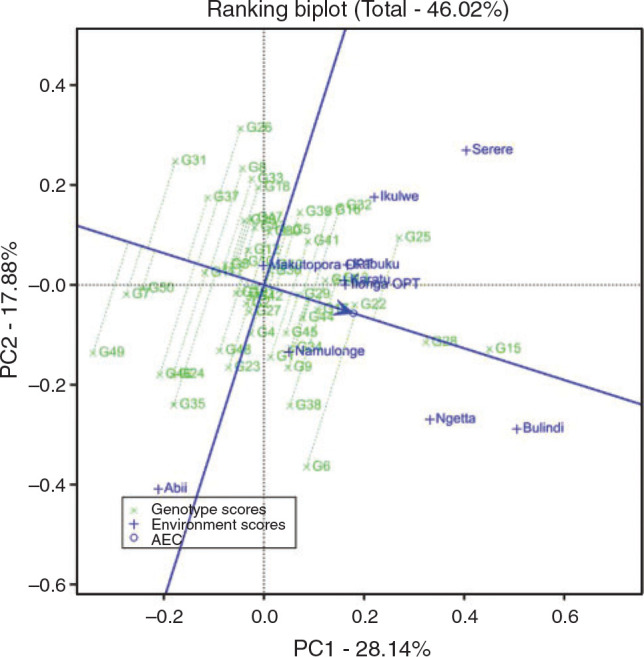
Mean vs stability view of the genotype main effect plus genotype × environment interaction biplot based on yield data of 50 maize varieties evaluated across 10 locations under optimum conditions in Uganda and Tanzania.

The mean vs stability view of the GGE biplot of yield data under drought was used to assess the stability of the 50 genotypes (Fig. 2c). The genotypes were ranked along the average tester coordinate (ATC) axis (abscissa), with an arrow pointing to a greater value based on their mean performance across all environments. The line with the arrow discriminates or separates entries with below-average means from those with above-average means. The average yield of the genotypes is approximated by the projections of their markers on the average tester axis. In the GGE biplot analysis, the ATC approximates the genotype contribution to GE, which is a measure of their instability. The stability of the genotypes is measured by their projection on the ATC axis; the longer the genotype’s projection, the less stable it is. Based on this, the top three stable genotypes under drought were CKDHH1081 (G9), CKDHH1148 (G22) and CKDHH1098 (G16) (Fig. 2c), and under optimum conditions, CKDHH0947 (G29), CKDHH1148 (G22) and CKDHH1097 (G15) (Fig. 3) were the most stable genotypes because they had a near zero projection onto the ATC axis. This implies that their ranking was highly consistent across locations. Conversely, genotype CKDHH1123 (G20) under drought stress, and CKDHH1075 (G6) under optimum moisture conditions, were the most unstable among the genotypes evaluated because they had longer projections away from the ATC axis than the other genotypes.

## Discussion

This study was conducted to examine the potential agronomic performance of drought-tolerant, doubled-haploids inbred lines developed and selected under managed drought stress in their different hybrid combinations across variable growing conditions in East Africa. There were significant differences for most traits, which demonstrated that selection could be made under both optimum conditions and random drought. Beyene *et al*. (2013), Adebayo and Menkir (2014) and Ertiro *et al*. (2017) reported differential responses of tropical maize hybrids under different environmental conditions. The non-existence of significant GE interaction effects under random drought suggested that testcross hybrids had consistent performance, which agrees with the work of Adebayo and Menkir (2014). The average grain yield of the top 15 experimental hybrids was higher than the best check under all management conditions, indicating that most of the experimental hybrids were superior to, and had greater stability than, the commercial checks. These results were consistent with the results of other authors who reported better tolerance to drought among new varieties (Beyene *et al*. 2013; Adebayo and Menkir 2014; Ertiro *et al*. 2017). However, the average grain-yield reduction for the experimental hybrids under random drought in our study was only 16% compared with the yield under optimum moisture conditions, indicating that the drought stress was low to moderate.

The doubled-haploid hybrids exhibited a wide-ranging variation in grain yield and other agronomic traits under both optimum-moisture and drought-stress environments. Similar observations were reported by Sserumaga *et al*. (2016) while examining performance and GE interactions of doubled-haploid hybrids in Uganda. This might imply that several factors could be affecting the performance of maize in the same or different environments.

Our results showed that the hybrids derived from doubled-haploid inbred lines outperformed the commercial hybrids for grain yield and other agronomic traits. Similar to the present study, Sserumaga *et al*. (2016) and Beyene *et al*. (2011) reported superiority in performance of doubled-haploid hybrids over the commercial checks in their studies. Under random drought stress, the highest yielding doubled haploid in the present work (CKDHH1090) had a 44.2% yield advantage over the best popular commercial hybrid, Com Check 3 (G48); however, under optimum environments, the highest yielding doubled haploid (CKDHH1097) had a 23% yield advantage over the best commercial hybrid, Com Check 1 (G46). This implied that the doubled-haploid hybrids were superior in performance to the commercially available hybrids. Therefore, the performance of the doubled-haploid hybrids indicated that the lines used in their development offered potential new sources for accelerating the breeding of high-yielding, drought-tolerant maize hybrids in similar tropical environments.

Under conditions of random drought and optimum moisture, the proportion of the total variance in grain yield attributed to the environment was high, whereas genotype and GE variances were relatively small. The broad-sense heritability for grain yield under drought was very low, which meant that selection for grain yield under this environmental setting led to low genetic gains. However, the broad-sense heritability for grain yield under optimum moisture conditions was higher (0.57) than under drought stress.

Genetic correlations among some locations were positive, indicating that the germplasm selected at any one of these locations could be used at the other, positively correlated locations. However, the reverse is true for those with negative correlations. The genetic correlations of grain yield under drought-stress and optimum-moisture environments were generally weak, which implied that grain yield was mediated mainly by the same set of genes that conditioned similar responses in the hybrids in the two contrasting environments. These results are similar to those reported for grain yield in maize grown under organic and conventional production systems (Lorenzana and Bernardo 2008). Although the correlation in that study was slightly higher than in our study, the results were different from those of Bänziger *et al*. (1997), who reported that different sets of genes controlled maize grain yield under low and high nitrogen environments. There was a low genetic correlation (*r*_g_ = 0.04) between some of the pairs of locations, implying that these environments were very different. Burdon (1977) pointed out that locations with low genetic correlations between them should be treated separately.

The stability and performance of different doubled-haploid hybrids were assessed by using GGE biplots to identify the best entries at each location and assess their stability. Emphasis was mainly on high grain yield under optimum moisture and improved yields under random drought, coupled with stable performance across sites with acceptable secondary traits. The most stable varieties in both environmental conditions were all derived from the doubled-haploid homozygous lines. These lines therefore contained favourable genes with additive effects resulting in heterosis (hybrid vigour). The fixation of favourable alleles in parental lines of the hybrids that performed well across stress environments contributes to the superior performance of hybrids (Ertiro *et al*. 2017). Hybrid CKDHH1098 (G16) ranked second best across all environments (6.82 t ha^−1^) and was the most stable hybrid evaluated. Therefore, it has the potential to be grown in a wide range of drought-prone environments in East Africa because of its broad adaptation. Two of the highest yielding varieties, CKDHH1090 (G14) and CKDHH1075 (G6), were not among the most stable, suggesting that these varieties have specific adaptation to some environments. These results are consistent with those of Badu-Apraku *et al*. (2012) and Makumbi *et al*. (2015), who identified high-yielding but unstable varieties in different, contrasting environments. This means that higher average yield indicated a higher response to favourable environments, but could result in lower environmental stability.

## Conclusion

This study showed that commercial varieties in the East African market are more vulnerable to drought and therefore less productive in various agro-ecologies in East Africa than the recent, new hybrids developed from the drought-tolerant doubled-haploid inbred lines through the WEMA public–private partnership. In terms of future projections, East Africa is more likely to be more vulnerable to drought if temperatures continue to increase. Results of the present study demonstrate that it is possible to have hybrids bred and released across East Africa and be recommended across different environmental conditions. This study also laid the foundation for exploiting GE interactions, not only to identify stable genotypes but also to classify environments into broader mega-environments, and to identify the most discriminating, high-yielding and stable environment for maize production in East Africa. Therefore, commercialisation of outstanding hybrids, such as CKDHH1098 (G16) identified in the present study, with high mean yield and stable performance across contrasting management conditions would contribute to enhancing maize productivity and yield stability for smallholder farmers.
