# Supplementary material for: Grain-yield stability among tropical maize hybrids derived from doubled-haploid inbred lines under random drought stress and optimum moisture conditions
Source: Crop Pasture Sci. 2018 Jul 4;69(11):691–712. doi: 10.1071/CP17348 (PMC7680931; doi:10.1071/CP17348)
Supplement: Supplementary file 1 [file CPS-69-07-691-s001.pdf]

10.1071/CP17348_AC
© CSIRO 2018

Supplementary Material: *Crop & Pasture Science*, 2018, 69, 691–702.

Grain-yield stability among tropical maize hybrids derived from doubled-haploid inbred lines under random drought stress and optimum moisture conditions

Julius Pyton Sserumaga[A,G], *Yoseph Beyene*[B], *Kiru Pillay*[C], *Alois Kullaya*[D], *Sylvester O. Oikeh*[E], *Stephen Mugo*[B], *Lewis Machida*[B], *Ismail Ngolinda*[F], *Godfrey Asea*[A], *Justin Ringo*[F], *Michael Otim*[A], *Grace Abalo*[A] *and Barnabas Kiula*[F]

[A]National Agricultural Research Organisation, National Crops Resources Research Institute, Namulonge, PO Box 7084 Kampala, Uganda.

[B]International Maize and Wheat Improvement Center (CIMMYT), ICRAF House, UN Avenue, Gigiri, Village Market, PO Box 1041-00621, Nairobi, Kenya.

[C]Monsanto, 2 Vermeulen Straat, Petit, 1512, South Africa.

[D]Mikocheni Agricultural Research Institute, PO Box 6226, Dar es Salaam, Tanzania.

[E]African Agricultural Technology Foundation (AATF), PO Box 30709-00100, Nairobi, Kenya.

[F]Ilonga Agricultural Research Institute, PO Box 33, Kilosa, Morogoro, Tanzania.

[G]Corresponding author. Email: j.serumaga@gmail.com

Supplementary Table 1: Description of genotypes used in the study and their source of origin

Genotype	Pedigree	Source	Derivative
G1	CKDHH1060	WEMA-CIMMYT	Derived from Doubled Haploid inbred lines
G2	CKDHH1066	WEMA-CIMMYT	Derived from Doubled Haploid inbred lines
G3	CKDHH1068	WEMA-CIMMYT	Derived from Doubled Haploid inbred lines
G4	CKDHH1070	WEMA-CIMMYT	Derived from Doubled Haploid inbred lines
G5	CKDHH1074	WEMA-CIMMYT	Derived from Doubled Haploid inbred lines
G6	CKDHH1075	WEMA-CIMMYT	Derived from Doubled Haploid inbred lines
G7	CKDHH1076	WEMA-CIMMYT	Derived from Doubled Haploid inbred lines
G8	CKDHH1077	WEMA-CIMMYT	Derived from Doubled Haploid inbred lines
G9	CKDHH1081	WEMA-CIMMYT	Derived from Doubled Haploid inbred lines
G10	CKDHH1078	WEMA-CIMMYT	Derived from Doubled Haploid inbred lines
G11	CKDHH1079	WEMA-CIMMYT	Derived from Doubled Haploid inbred lines
G12	CKDHH1088	WEMA-CIMMYT	Derived from Doubled Haploid inbred lines
G13	CKDHH1089	WEMA-CIMMYT	Derived from Doubled Haploid inbred lines
G14	CKDHH1090	WEMA-CIMMYT	Derived from Doubled Haploid inbred lines
G15	CKDHH1097	WEMA-CIMMYT	Derived from Doubled Haploid inbred lines
G16	CKDHH1098	WEMA-CIMMYT	Derived from Doubled Haploid inbred lines
G17	CKDHH1100	WEMA-CIMMYT	Derived from Doubled Haploid inbred lines
G18	CKDHH1102	WEMA-CIMMYT	Derived from Doubled Haploid inbred lines
G19	CKDHH1106	WEMA-CIMMYT	Derived from Doubled Haploid inbred lines
G20	CKDHH1123	WEMA-CIMMYT	Derived from Doubled Haploid inbred lines
G21	CKDHH1124	WEMA-CIMMYT	Derived from Doubled Haploid inbred lines
G22	CKDHH1148	WEMA-CIMMYT	Derived from Doubled Haploid inbred lines
G23	CKDHH1132	WEMA-CIMMYT	Derived from Doubled Haploid inbred lines
G24	CKDHH1134	WEMA-CIMMYT	Derived from Doubled Haploid inbred lines
G25	CKDHH1141	WEMA-CIMMYT	Derived from Doubled Haploid inbred lines
G26	CKDHH1149	WEMA-CIMMYT	Derived from Doubled Haploid inbred lines
G27	CKDHH1145	WEMA-CIMMYT	Derived from Doubled Haploid inbred lines
G28	CKDHH1146	WEMA-CIMMYT	Derived from Doubled Haploid inbred lines
G29	CKDHH0947	WEMA-CIMMYT	Derived from Doubled Haploid inbred lines
G30	CKDHH0954	WEMA-CIMMYT	Derived from Doubled Haploid inbred lines
G31	CKDHH0957	WEMA-CIMMYT	Derived from Doubled Haploid inbred lines
G32	CKDHH0959	WEMA-CIMMYT	Derived from Doubled Haploid inbred lines
G33	CKDHH0960	WEMA-CIMMYT	Derived from Doubled Haploid inbred lines
G34	CKDHH0969	WEMA-CIMMYT	Derived from Doubled Haploid inbred lines
G35	CKDHH1001	WEMA-CIMMYT	Derived from Doubled Haploid inbred lines
G36	CKDHH1003	WEMA-CIMMYT	Derived from Doubled Haploid inbred lines
G37	CKDHH1004	WEMA-CIMMYT	Derived from Doubled Haploid inbred lines
G38	CKDHH1005	WEMA-CIMMYT	Derived from Doubled Haploid inbred lines
G39	CKDHH1007	WEMA-CIMMYT	Derived from Doubled Haploid inbred lines
G40	CKDHH1024	WEMA-CIMMYT	Derived from Doubled Haploid inbred lines
G41	CKDHH1131	WEMA-CIMMYT	Derived from Doubled Haploid inbred lines
G42	CKDHH1143	WEMA-CIMMYT	Derived from Doubled Haploid inbred lines
G43	CKDHH1044	WEMA-CIMMYT	Derived from Doubled Haploid inbred lines
G44	CZH0616	WEMA-CIMMYT	Derived from Pedigree bred inbred lines
G45	WE1101	WEMA-CIMMYT	Derived from Pedigree bred inbred lines
G46	Com Check 1	Seed company	Derived from Pedigree bred inbred lines
G47	Com Check 2	Seed company	Derived from Pedigree bred inbred lines
G48	Com Check 3	Seed company	Derived from Pedigree bred inbred lines
G49	Local Check1	NARS	Derived from Pedigree bred inbred lines
G50	Local Check2	NARS	Derived from Pedigree bred inbred lines

Supplementary Table 2: Characteristics description of the sites where DH testcross hybrids were evaluated during the study period (2014)

Site	Country	Management	Latitude	Longitude	Elevation (masl)	Mean Rainfall (mm)	Temperature (ºC)		Soil Type
							Min	Max	
Namulonge	Uganda	Optimum rain-fed	$0^0$36'N'	$32^0$36'E'	1150	1270	16	28	Sandy clay loam
Serere	Uganda	Optimum rain-fed	$1^0$31'N	$33^0$27'E'	1080	1419	19	31	Sandy clay loams and black clays
Bulindi	Uganda	Optimum rain-fed	$0^0$16'N	$32^0$52'E'	1144	1338	19	29	Sandy loam
Ngetta	Uganda	Optimum rain-fed	$2^0$16'N'	$32^0$52'E	1300	1483	19	29	Sandy loam
Masaka	Uganda	Managed Drought	$0^0$18'S'	$31^0$39'E'	1220	1174	14	28	Red laterite, sandy loam
Abi	Uganda	Optimum rain-fed	$3^0$05'N'	$30^0$57'E'	2854	1250	16	32	Ferralitic and sand loams.
Ikulwe	Uganda	Optimum rain-fed	$0^0$26'N'	$33^0$28'E'	1170	1345	15	29	
Kasese	Uganda	Optimum rain-fed	$0^0$10'S'	$30^0$04'E'	960	1200	18	31	Peaty sands and Clay
Ilonga	Tanzania	Optimum rain-fed and Managed Drought	$6^0$47'S'	$37^0$02'E'	506	1059	16	36	Clay Loam
Kabuku	Tanzania	Optimum rain-fed	$5^0$49'S'	$38^0$48'E'	401	921	20	37	Red soils
Karatu	Tanzania	Optimum rain-fed	$3^0$34'S'	$35^0$39'E'	1522	905	15	24	Black Clays
Makutupora	Tanzania	Optimum rain-fed and Managed Drought	$5^0$58'S'	$35^0$46'E'	1086	596	14	32	Sandy Loam

masl: metres above sea level

Supplementary Table 3: Mean performance of top 15 hybrids, and last 5 hybrids across 13 environments in Uganda and Tanzania.

The ranking was based on yield.

Rank	No.	Genotype	Grain Yield (GY)	Days to Anthesis (AD)	Anthesis silking interval (ASI)	Husk Cover (HC)	Ear Aspect (EA)	Ear height (EH)	Ear Per Plant (EPP)	Ear Position (EP)	Grain Moisture (MOI)	Plant Aspect (PA)	Plant Height (PH)
			(t ha-1)	(days)	(days)	(%)	(1-5)	(cm)	(no. of ears)	(cm)	(%)	(1-5)	(cm)
Top													
1	G14	CKDHH1090	7.01	63.39	1.83	4.38	2.43	95.43	0.47	0.97	18.24	2.74	203.09
2	G16	CKDHH1098	6.82	64.60	2.56	3.88	2.78	112.21	0.54	0.91	18.87	2.78	200.81
3	G6	CKDHH1075	6.47	64.66	2.68	4.29	2.89	103.19	0.50	0.97	17.24	2.82	204.67
4	G43	CKDHH1044	6.47	64.09	2.43	5.22	2.89	96.46	0.48	0.91	18.37	2.65	198.60
5	G32	CKDHH0959	6.44	63.71	2.41	5.25	2.91	103.56	0.51	1.00	18.47	2.71	198.12
6	G30	CKDHH0954	6.30	65.62	2.63	4.97	2.79	94.89	0.47	0.89	18.20	2.72	195.09
7	G44	CZH0616	6.29	60.99	3.37	4.81	2.39	101.61	0.50	0.91	16.63	2.72	200.03
8	G22	CKDHH1148	6.26	64.80	2.59	5.47	2.69	107.54	0.50	0.92	17.92	2.76	210.22
9	G23	CKDHH1132	6.12	67.22	2.24	6.82	3.12	102.04	0.65	0.90	17.81	2.84	173.03
10	G33	CKDHH0960	6.10	67.63	3.40	6.49	3.22	104.77	0.51	0.86	18.81	3.23	206.67
11	G9	CKDHH1081	6.04	62.73	2.29	6.73	2.35	107.11	0.49	0.96	16.37	2.59	214.57
12	G3	CKDHH1068	5.99	64.92	2.09	9.00	2.86	97.69	0.48	0.97	17.41	2.97	201.71
13	G4	CKDHH1070	5.91	63.75	3.33	6.77	2.90	103.80	0.49	0.91	18.04	2.79	209.29
14	G19	CKDHH1106	5.87	63.23	2.96	5.36	2.45	96.18	0.45	0.91	18.28	2.58	208.47
15	G39	CKDHH1007	5.86	64.45	2.21	7.81	2.69	106.96	0.55	0.95	18.18	2.57	194.67
**Last**													
45	G42	CKDHH1143	5.03	65.74	2.54	6.14	2.92	98.73	0.50	0.79	17.71	2.84	201.52
46	G12	CKDHH1088	4.90	65.45	2.77	5.13	2.52	111.67	0.54	0.84	18.53	3.05	210.46
47	G27	CKDHH1145	4.89	65.34	3.40	10.96	2.58	102.29	0.55	0.99	18.54	2.47	199.62
48	G48	Com Check 3	4.86	64.58	3.02	8.82	2.65	103.37	0.56	0.79	16.83	2.78	187.18
49	G10	CKDHH1078	4.85	64.52	2.93	3.64	2.44	102.97	0.47	0.93	17.52	2.95	218.48
50	G24	CKDHH1134	4.69	68.62	2.04	8.06	2.58	97.04	0.51	0.87	18.10	2.84	190.67

**Mean**	**5.64**	**65.18**	**2.71**	**6.05**	**2.68**	**103.71**	**0.51**	**0.90**	**17.98**	**2.81**	**203.35**
**Minimum**	**4.69**	**60.99**	**1.83**	**3.64**	**1.91**	**94.48**	**0.45**	**0.78**	**16.19**	**2.47**	**173.03**
**Maximum**	**7.01**	**68.62**	**4.14**	**10.96**	**3.22**	**114.88**	**0.65**	**1.00**	**19.31**	**3.23**	**234.13**
**LSD0.05**	**1.14**	**3.65**	**1.01**	**3.18**	**0.58**	**15.90**	**0.12**	**0.12**	**1.51**	**0.39**	**28.19**

Supplementary Table 4a: Genetic correlations for grain yield of 43 testcross hybrids, 2 internal WEMA hybrid checks, 3 commercial hybrids and 2 local hybrid checks across locations with drought conditions in Uganda and Tanzania

	Makutupora DT	Ilonga DT
Ilonga DT	0.16	
Masaka	0.58	0.13

Supplementary Table 4b: Genetic correlations for grain yield of 43 testcross hybrids, 2 internal WEMA hybrid checks, 3 commercial hybrids and 2 local hybrid checks across locations with optimal conditions in Uganda and Tanzania

	Abii	Bulindi	Ikulwe	IlongaOPT	Kabuku	MakutoporaOPT	Namulonge	Ngetta
Abii								
Bulindi	-0.16							
Ikulwe	-0.54	0.07						
IlongaOPT	-0.24	0.09	0.31					
Kabuku	0.18	0.19	0.19	0.22				
MakutoporaOPT	0.10	-0.06	-0.06	-0.13	-0.01			
Namulonge	-0.07	0.24	-0.09	0.02	-0.13	0.06		
Ngetta	-0.26	0.39	0.18	0.32	0.13	-0.10	0.24	
Serere	-0.21	0.26	0.47	0.23	0.30*	0.09	-0.23	0.15

Supplementary Table 4c: Genetic correlations for grain yield of 43 Doubled haploid (DH) testcross hybrids, 2 internal WEMA hybrid checks, 3 commercial hybrids and 2 local hybrid checks across locations in Uganda and Tanzania

	Abii	Bulindi	Ikulwe	Ilonga_DT	Ilonga_OPT	Kabuku	Makutopora_DT	Makutopora_OP	Masaka	Namulonge	Ngetta
Abii											
Bulindi	-0.16										
Ikulwe	-0.54	0.07									
Ilonga_DT	-0.19	-0.04	0.19								
Ilonga_OPT	-0.24	0.09	0.32	0.29							
Kabuku	0.18	0.19	0.19	0.16	0.22						
Makutopora_DT	-0.06	-0.03	0.13	-0.13	0.03	0.21					
Makutopora_OP	0.10	-0.06	-0.06	0.24	-0.13	-0.01	0.04				
Masaka	0.01	0.24	0.01	0.13	-0.01	0.22	0.58	0.08			
Namulonge	-0.07	0.24	-0.09	0.10	0.02	-0.13	0.09	0.06	0.39		
Ngetta	-0.26	0.39	0.18	0.06	0.32	0.13	0.13	-0.10	0.13	0.24	
Serere	-0.21	0.26	0.47	0.20	0.23	0.30	0.15	0.09	0.11	-0.23	0.15
